# Complete Genome Sequence of SS52, a Strain of Escherichia coli O157:H7 Recovered from Supershedder Cattle

**DOI:** 10.1128/genomeA.01569-14

**Published:** 2015-03-19

**Authors:** Robab Katani, Rebecca Cote, Juan Antonio Raygoza Garay, Lingling Li, Terrance M. Arthur, Chitrita DebRoy, Michael M. Mwangi, Vivek Kapur

**Affiliations:** a Department of Veterinary and Biomedical Science, Pennsylvania State University, University Park, Pennsylvania, USA; b The Huck Institutes of the Life Sciences, Pennsylvania State University, University Park, Pennsylvania, USA; c Roman L. Hruska U.S. Meat Animal Research Center, Agricultural Research Service, U.S. Department of Agriculture, Clay Center, Nebraska, USA; d *E*. *coli* Reference Center, Pennsylvania State University, University Park, Pennsylvania, USA; e Department of Biochemistry and Molecular Biology, Pennsylvania State University, University Park, Pennsylvania, USA

## Abstract

Shiga toxin-producing Escherichia coli O157:H7 causes foodborne infections, and cattle are the primary reservoir. Some animals, known as supershedders, excrete orders of magnitude more E. coli O157:H7 in the feces than normal. Here, we report the complete genome sequence of the SS52 supershedder strain of E. coli O157:H7.

## GENOME ANNOUNCEMENT

Shiga toxin-producing Escherichia coli O157:H7 (O157) is a zoonotic foodborne pathogen of major public health concern that results in considerable human intestinal and extra-intestinal illness (1–3). O157 is primarily transmitted to humans through the consumption of contaminated food or water or through exposure to infected animals (3).

Asymptomatic cattle are the primary source of human infection, and O157 colonizes the terminal recto-anal junction (RAJ) of infected animals that typically shed the bacteria at 10 to 100 CFU/g of feces, contributing to environmental contamination, transmission of the pathogenic bacteria, and ultimately contamination of the food supply. A subgroup of cattle, termed as “supershedders,” excretes O157 at levels ≥10^4^ CFU/g of feces (4, 5). Several epidemiological studies have indicated that although the number of supershedder animals on farms is often less than 10%, these animals are responsible for 99% of the bacteria shed into the environment (6, 7). Experimental evidence has shown that swabbing the RAJ directly with O157 results in infection and carriage of the bacteria similar to what occurs in cattle that have been naturally infected, indicating the importance of the RAJ in mimicking natural infections (8). However, the molecular mechanisms that contribute to the adherence and colonization of O157, especially supershedder (SS) strains, to the bovine RAJ remains elusive.

We recently characterized the first complete genome sequence and phenotypic characteristics of a supershedder strain of O157, SS17 (accession no. CP008805 [9]). The results suggest that supershedder isolates have distinctive genomic and phenotypic features, including a novel hyperaggregative phenotype on bovine rectal epithelial cells (9). In order to further elucidate genomic factors that might contribute to the adherence and colonization of SS strains to the bovine RAJ, we report the complete genome sequence of a second supershedder strain of O157, SS52, part of a large collection of supershedder isolates recovered from cattle in the Midwestern United States (10).

Purified genomic SS52 DNA was processed for whole-genome shotgun sequencing using Ion Torrent PGM technology (Life Technologies, Grand Island, NY) (11). Using a 318 sequencing chip and mate-pair sequencing, a total of 5.4 M reads with an average length of 169 bases was obtained with 168.4-fold coverage. Both *de novo* and reference-guided assemblies were performed using DNASTAR SeqMan NGen v. 11.0.0 and Lasergene Suite (Madison, WI). The genome was closed with manual primer walking and Sanger sequencing. The final assembly was anchored to an optical map generated by OpGen, Inc. (Gaithersburg, MD) (12). The genome was annotated using Rapid Annotation using Subsystem Technology (13), followed by manual curation in Artemis (14). The results show that SS52 has a chromosome size of 5,488,700 bp with 5,632 open reading frames and one plasmid, pO157 (94,730 bp). A total of 3,106 and 801 single nucleotide polymorphisms (SNPs) were identified in SS52 as compared to the EDL933 and SS17 genomes, respectively. Further studies are needed to explore the role, if any, of these SNPs in contributing to the supershedder phenotype.

### Nucleotide sequence accession numbers.

The whole-genome sequence of SS52 has been deposited at DDBJ/ENA/GenBank with the accession numbers CP010304 (SS52) and CP010305 (SS52_pO157).
